# Integrative Field-Based Health and Performance Research: A Narrative Review on Experimental Methods and Logistics to Conduct Competition and Training Camp Studies in Athletes

**DOI:** 10.1007/s40279-025-02227-0

**Published:** 2025-04-21

**Authors:** Trent Stellingwerff, Louise M. Burke, Hannah G. Caldwell, Robert J. Gathercole, Chris J. McNeil, Christopher Napier, Sarah A. Purcell, Susan Boegman, Elizabeth Johnson, Sharleen D. Hoar, Alexandra M. Coates, Erica V. Bennett, Alannah K. A. McKay, Ida. A. Heikura, Michael J. Joyner, Jamie F. Burr

**Affiliations:** 1 Canadian Sport Institute-Pacific, Victoria, British Columbia Canada; 2 Exercise Science, Physical and Health Education, University of Victoria, Victoria, British Columbia Canada; 3 School of Kinesiology, The University of British Columbia, Vancouver, British Columbia Canada; 4 Mary Mackillop Institute for Health Research, Australian Catholic University, Melbourne, Victoria Australia; 5 School of Health and Exercise Sciences, The University of British Columbia, Okanagan Campus, Kelowna, British Columbia Canada; 6 The August Krogh Section for Human Physiology, Department of Nutrition, Exercise and Sports, University of Copenhagen, Copenhagen, Denmark; 7 Product Innovation Team, Lululemon Athletica, Vancouver, British Columbia Canada; 8 Department of Biomedical Physiology and Kinesiology, Simon Fraser University, Vancouver, British Columbia Canada; 9 Centre for Chronic Disease Prevention and Management, Southern Medical Program, Department of Medicine, The University of British Columbia, Kelowna, British Columbia Canada; 10 Department of Anesthesiology and Perioperative Medicine, Mayo Clinic, Rochester, Minnesota USA; 11 Human Health and Nutritional Sciences, University of Guelph, Guelph, Ontario Canada

## Abstract

Field-based sport research involves studies that collect data from athletes and/or teams during competition and/or their daily training environments. Over the last decade, sport-specific field-based research projects have significantly increased in number and complexity, partially owing to the further development of more portable measurement equipment (e.g., indirect calorimetry, desktop blood/gas analyzers, portable laboratories, etc.) and/or wearable or consumable technologies (e.g., smart watches, sensors, core temp pills, etc.). However, given these rapid advances and novelty, challenges remain in the validity and applicability of these devices. Unfortunately, there are no global ethical or best-practice standards for the use of portable devices and/or wearables in sport; however, this review will outline various opportunities and challenges. Many decision trade-offs are required when designing field-based research studies to balance gold-standard scientific rigor and strict research control with highly applied, but less-controlled, “real-world” conditions. To our knowledge, there are no narrative reviews that take a wholistic view of the logistical and methodological considerations of field-based research in athletes. Accordingly, this review takes a multi-disciplinary methodological approach (physiological, nutritional/energetic, biomechanical, musculoskeletal, cognitive, and psychosocial factors), along with the logistical considerations involved in project planning, research design, and ethics of field-based research with elite athletes and/or teams. We also provide practical guidance for characterizing the extreme demands of elite training and competition to support research that ultimately catalyzes improved understanding of the limits of human capacity. We hope this review can serve as a practical guide for researchers undertaking elite athlete field-based research.

**Supplementary Information:**

The online version contains supplementary material available at 10.1007/s40279-025-02227-0.

## Key Summary Points

**Table:** 

Elite athletes, teams, and coaches are most interested in research findings with high ecological validity that can be directly applied to improving health and performance in their sport—appreciating that the limits of human performance happen in the field, and not in the laboratory.
Most field-based studies are integrated into larger training camps or competitions, requiring the management of complex collaborations and partners, which can include athletes, teams, coaches, sport organizations, researchers, event organizers, academic institutions, and medical staff, as well as sport governing bodies, anti-doping agencies, funding agencies, and corporate and host communities.
The ideal scientific design and implementation of field-based research studies involving competition and/or training in elite athletes/teams requires: (1) researchers who have intimate knowledge of a sport/event; (2) the trust of the athletes and coaches; (3) exceptional logistical and organizational skills; and (4) rigorous scientific expertise to apply the various field-based methodologies.
The context and goals of a field-based research project should inform the choice of data collection method(s), as each approach to assessing physiological, nutritional/energetic, biomechanical, musculoskeletal, cognitive, and psychosocial factors has various logistical and compliance considerations independently and collectively as a research program.

## Introduction

The ultimate limits of human performance, and by extension the limits of physiological, biomechanical, and/or psychological capacities, occur in the field of competition—not in the laboratory. Naturally, athletes and coaches tend to be most interested in scientific findings that demonstrate high ecological validity [1] or direct application within their sport or event. Conversely, the blind pursuit of scientifically assessing “real-world conditions” (highest ecological validity), without appreciating methodological limitations, can cloud mechanistic understanding or causative outcomes of applied field-based studies [2]. Ideally, there is an integrated consideration in study design and methodological decisions between gold-standard scientific rigor versus highly applied, but less-controlled, field-based research pursuits. In a distinguished lecture, Dr. Sandra Hunter eloquently reviewed this dichotomy, concluding “that an integrated approach encompassing analysis of real-world data and experimental studies provides unique insights to the biological and sociocultural factors attributed to the limits of performance” [3].

Within this review, we define “field-based sport research” as any study designed to generate data from athlete(s) and/or teams undertaking competition (including competition results) and/or any training and/or medical/therapy interventions in their typical daily training or competition environments. Furthermore, from a sport (athlete/team/coach/practitioner) lens, we define “sport ecological validity” as having a study design and outcomes that are easily applied to the unique athlete/team populations [4] within a specific sport or event.

By definition, elite/world-class athletes (Tiers 4–5, defined by McKay et al. [4]) are uniquely suited to field-based investigations. World-class (Tier 5) athletes represent only < 0.00006% of the global population, and many elite athletes undertake 30 + h/week of training, which equates to extreme exercise energy expenditures (EEE) [5–11], potentially beyond what humans have evolved to chronically (over weeks to months) expend [12]. Moreover, cross-sectional descriptive field-based studies are often the only feasible research design for elite and world-class athletes/teams, as these are the studies in which they are typically most willing to participate. Therefore, field-based research design needs to consider validated methodological approaches that are also the least obtrusive to the athletes/teams.

Seminal field-based research has a long history, including occupation studies (e.g., heat acclimation in miners [13]), military investigations (e.g., Harvard Fatigue Lab’s contributions to WWII research [14]), and altitude expeditions (e.g., Dr. Griffith Pugh’s research on Everest in the 1950s [15]). Field-based research in sport has an even longer history, dating back over a century, including studies on blood glucose and carbohydrate intake at the Boston Marathon and A.V. Hill’s modeling of world record times and critical speed [16, 17]. The current authors additionally have extensive sport field-based publications [18–21]. Although there have been several altitude expedition field-based methodological/logistical reviews [22, 23], and papers capturing domain specific methodological considerations of sport and exercise field-based research [18, 19, 24–27], we are unaware of any prior comprehensive methodological and logistical reviews that have attempted to capture all aspects of applied athlete and sport field-based research design.

**Fig. 1:**
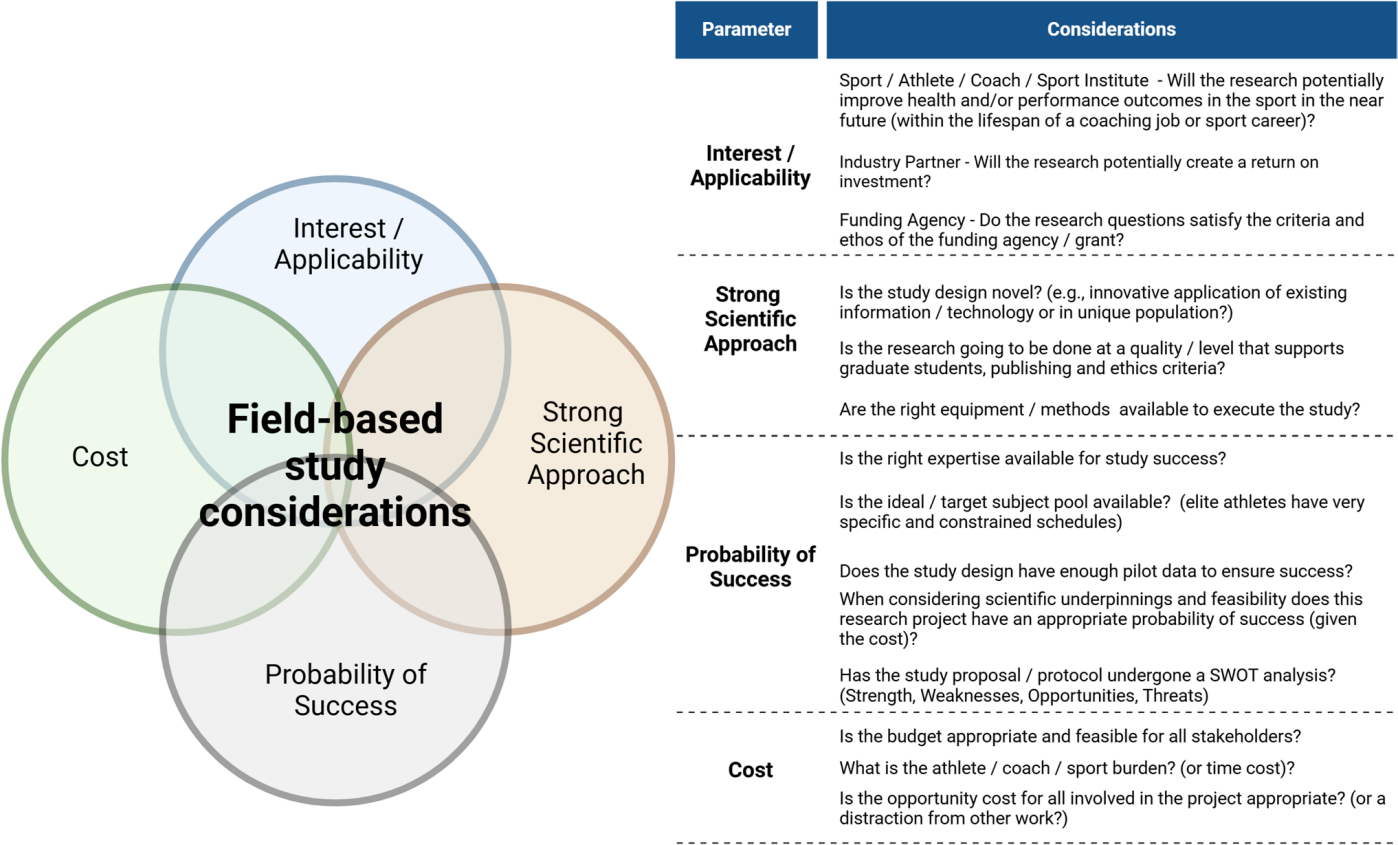
Methodological and logistical study design considerations prior to field-based data collection

Therefore, the primary purpose of this review is to integrate a multi-discipline approach in characterizing the logistical and methodological considerations for field-based sport (athletes and teams) research. This review includes subsections on physiological, nutritional/energetic, biomechanical, musculoskeletal (MSK), cognitive, and psychosocial factors, as well as logistical and methodological considerations, including project planning, research design, ethics, and specific considerations for female athletes. Our review authors feature a wide range of expertise across each subsection, and each undertook an extensive general search of literature, specific to research methods and logistics, to support each section, which resulted in extensive supporting citations. We emphasize aspects of applicability, scientific underpinnings, feasibility, availability (expertise, equipment, and participants), and probability of success and cost (money, time, athlete, and opportunity cost; Fig. 1). We believe this to be timely, given the recent advances in “wearable” technologies that easily support field-based research, while also appreciating the strengths and limitations of these technologies [24]. This review aims to serve as a practical “A to Z” guide for researchers undertaking field-based research—from study inception to in-field execution.

## Research Project Planning, Research Design, Ethics Approval, and Methodological Considerations

This section offers a framework for planning and executing complex field-based projects that marry scientific rigor with practical feasibility to ensure research that is impactful and relevant to the real-world athlete experiences. Many field-based studies are integrated into larger training camps or competitions, requiring the management of complex collaborations and partners. This can include: athletes, coaches, sport organizations, researchers, event organizers, academic institutions, and medical staff, as well as sport governing bodies, anti-doping agencies, funding agencies, corporate sponsors (for the event, equipment, or individual athletes), and host communities (e.g., local or national governments).

### Initial Feasibility Review and Defining Objectives

As the initial concept is developed, a thorough feasibility review should be conducted to establish the goals/requirements of all collaborating partners [i.e., stakeholders (Table 1; Fig. 1)], as well as key research and nonresearch outcomes. The likelihood of success, weaknesses, or threats, as uniquely defined by each collaborating partner, should be assessed across the entire project. It is important to note that partner goals can potentially not be scientific in nature or specific to the research team’s objectives. The feasibility review can also serve as an initial “go or no-go” step, to ensure project viability for all collaborators involved, and should include subject matter experts that can address unique features (i.e., research versus performance logistics [28]). In addition, a successful feasibility review will be iterative in nature as potential threats and solutions are identified. Communication mechanisms, such as meeting cadence, should be clearly defined to meet the needs of all collaborators participating.

**Table 1:** Overview of the collaborator goals and requirements for field-based study planning and success

Collaborating partner (s)	Typical goals/requirements for in-field study success
Athletes/coaches/sport governing body (national or international)	Will the research question improve performance, decrease injury or illness outcomes, and/or enhance sport decision-making, either now or in the future?
	Is the proposed research intervention practical and feasible to implement within the specific athlete and sport context (e.g., within the rules of the sport)?
	What is the study participation ‘burden’? Will athletes be able to fully train and/or maximally perform within the study protocol?
	Is the intervention financially and logistically feasible?
Corporate/industry/equipment partners [sponsor(s) of the research, event, specific equipment or individual athlete sponsor]	Will the evidence derived have a potential financial return on investment? (e.g., new product development, validation of product/equipment, product testing/piloting with athletes, increased competitive knowledge, and/or enhanced brand credibility and awareness?)
	Will event or corporate sponsorship lead to real or perceived bias in study design or outcomes (e.g. selection of control groups, or interventions that are, or perceived to be, in the sponsor’s favor)?
	Do participating athlete sponsorship deals dictate or prohibit the use of certain equipment/clothing (i.e., wearables), methodological protocols and/or research designs?
	Does a sponsoring partner (e.g., equipment, facilities etc.) require the use of equipment or branding? What research outcomes are required to support the sponsoring partners?
	Are scientific equipment partners stable and engaged throughout the study's duration (e.g., financial viability, product support) and can they provide unprocessed data in a timely manner (e.g., to support “live” data monitoring)?
Anti-doping or International Sport Federations/Agencies (national/international)	Does the research design interfere with any of the World Anti-Doping Code (WADA) requirements for its elite athlete testing programs? (i.e., the required availability for doping control activities that may occur during training or the event)
	Does the research intervention contravene the WADA doping code or the rules of the individual sport?
Competition or training camp host community	Is there a local community champion that can advocate and help with local research infrastructure and logistics?
	Are there any social, brand or environmental impacts of the research project that the community needs to be aware of, or involved with?
	Is there an emergency action plan (EAP) developed in conjunction with/vetted by local authorities and service providers in the community?
	For internationally located projects, have you developed relationships with community ‘champions’ that understand the international laws and requirements? This may include having to hire a language translator before and during the study (both for researchers, but also for international athletes)
Ethics Institutional Review Boards (IRB)/Academic Institutions/Funding Agencies	Are the research design and methods rigorous enough to ensure academic and scientific quality? Are they sufficient for academic programs (e.g., undergraduate to post-graduate studies) in cases where students/trainees are embedded in the project?
	Are research questions/scope aligned with the scope and ethos of academic institutions and funding agencies?
	Are research questions and methods ethically appropriate, with ethics review board approval from the parent and/or local academic institutions? (see more in Table 2)
Event (competition or training camp) medical support	Does the research protocol / plan have a well-vetted emergency action plan (EAP), that is co-developed with medical staff, and integrated and shared with all involved staff?
	Will the research protocols impact any medical support and/or vice versa? Are there expectations of the medical team to be involved with the research? What research briefing do the medical team need to optimize their role at the event?
	If appropriate to the study aims, can the event medical team be integrated into the research team?

### Research Design and Methodological Considerations within the Overall Field-Based Project

#### Research Project Personnel Roles

When assembling the research team, it is important to prioritize personnel with field-based and athlete research experience, a shared enthusiasm for the project’s goals, and a commitment toward collaborative work. Clear roles and responsibilities should be established. For example, project leaders (or sub-team leaders) are not only accountable for the project’s success but also may lead in securing the budget, managing challenges and logistics, and ensuring effective ongoing communication with all partners. Incorporating a mix of trainees, early-career researchers, and specific experts can enhance problem-solving capabilities and foster innovative approaches while creating significant opportunities for researchers starting their careers. It is also recommended that a proactive publication plan that clearly defines roles, expectations, and outcomes across the research team, and each anticipated paper, is developed prior to project implementation (example in Supplementary Material Table 1). However, part of this publication plan may sometimes feature publication “embargos” depending on the requirements of various collaborators (Table 1), especially as it pertains to potential competitive advantage outcomes for teams or federations.

#### Study Research Design: Balancing Validity, Practicality, and Athlete/Team Acceptability

Research designs and methodologies should be selected to strike a balance between the constraints of a field setting and the need to collect valid and reliable data. Field-based studies are characterized by their relevance to real-world conditions and ability to provide insights that are directly applicable to practice [29]. Many field-based studies tend to implement descriptive cross-sectional designs and often do not meet the scientific rigor of randomized controlled trials (RCTs). That said, RCTs can also lack generalizability, suffer from randomization and selection biases, and impose very high costs and ethical concerns around treatment or nontreatment outcomes [30, 31]. For field-based research in an elite sport environment, the design must prioritize robust, validated, and practical methodologies that are also minimally intrusive to athletes [18, 28].

Although tools such as Consolidated Standards Of Reporting Trials (CONSORT [32]) or Proper Report of Evidence in Sport and Exercise Nutrition Trials (PRESENT [33]) have been developed as author writing/preparation guidelines prior to scientific journal submissions, we also recommend their use a priori during the study development phase to aid in study design and methodological decisions.

Field-based research is often the only feasible research design for the involvement of Tier 4 and 5 elite/world-class athletes [4]. This level of athlete may only participate if their primary goal—optimal performance—is respected, while the advancement of scientific research is positioned as a secondary objective. Early engagement with these athletes and their coaches during the project feasibility phase can help identify methodological and practical challenges that may not be immediately apparent to researchers. This also promotes enhanced engagement among the athletes, coaches, and research team.

#### Statistical Significance Versus Elite Competition Significance

High-stakes competition results provide the most ideal real-world data for understanding the boundaries of human performance. However, we must also recognize that the tiny margins of winning in elite sport, which are highly relevant in terms of “competitive differences,” may be irrelevant when considered according to typical “statistical differences”[34]. It is beyond the scope of this paper to undertake an in-depth discussion of applied sport statistical approaches, which has recently been reviewed [35, 36]. Instead, we will focus on the challenging reality of interpreting field-based performance outcomes.

An example of field-based competitive significance could be from the 2011 Tour de France, which had a winning time of 86 h 12 min and 22 s, with 10th place being only 0.19% behind [~ 10 min difference over 86 h of racing, which is a massive competitive difference (1st to 10th place), but a tiny numerical/statistical difference]. For example, a sample-size power calculation to detect a 0.19% difference would require thousands of athletes to demonstrate this tiny performance difference, which, aside from practical considerations, is not even possible when the study population is world-class/elite athletes. Statistics aside, it is important to note that many coaches and athletes realize that a singular evidence-based performance intervention with an athlete within a complex competitive environment that is statistically significant is never certain. Instead, coaches and athletes/teams are mainly interested in the *possibility* of tiny performance margins as long as the positive performance effect appears to be consistent, the risk of an adverse or negative outcome is relatively low, and the opportunity and/or monetary cost is not prohibitive.

Field-based and laboratory performance studies of world-class/elite athletes have consistently demonstrated greater performance precision [smaller % coefficient of variation (CV) in repeated performance tests] in these athletes compared with recreational or national-class athletes [37]. However, owing to an ultimate performance ceiling, world-class/elite athletes also have smaller effect sizes to a given evidence-based performance intervention (e.g., trained athletes have ~ 3–4 times lower performance effect size outcomes to sodium bicarbonate supplementation than untrained subjects [38]). Given this, in elite athlete cohorts, it is important to understand a given athlete’s or team’s historical performance variability and progression, and ideally have repeated measures along with the coach’s realistic performance expectations, to guide whether a small performance change may be relevant. For example, in a recent performance-based pre–post study design in Olympic rowers [39], we used each individual athlete’s own normal performance variation [between past 2000 m rowing time-trial (TT)] and showed that almost all athletes were faster post intervention (18 of 20 improved pre to post TT; mean TT improvement, female: ~ 1.1 ± 1.2%, male: ~ 1.2 ± 0.5%). In addition, almost all athletes experienced a greater change in TT performance than would have been expected from historical data (normal % CV TT, female: ~ 0.3%, male: ~ 0.5%), which was highly relevant to these athletes and coaches. Ideally, study designs try to account for, and minimize, placebo effects; however, real-world performance is a complex mosaic of potentially hundreds of factors. These factors include all important relationships among athletes, coaches, and sport scientists, where you want to maximize “belief effects” but minimize cost and risk. This was nicely summarized in an editorial titled “Lying to Win—Placebos and Sport Science” [40].

#### Unique Considerations for Ethical Approval for Field-Based Research

Research-based ethics approval is rarely a requirement for athletic competition itself since competition is regulated through sport policy/rules, World Anti-Doping Agency (WADA) requirements for elite athletes [41], and professional player associations/unions. However, an institutional review board (IRB) must provide ethical approval for all human research projects, whether it occurs during competition or any other context, according to the 2013 World Medical Association Declaration of Helsinki [42, 43]. Researchers may need to submit for IRB approval for each location considered for their field-based project. The IRB evaluates aspects including athlete safety, confidentiality, conflict of interest, governance, research design (especially pertaining to placebo and/or deception), sample size, athlete burden [44], social media engagement (e.g., recruitment), data management, and informed consent [42, 43, 45]. Ethical research principles are consistently updated to cover ever-changing technological advances (e.g., data security/confidentiality regarding open-access data and data collection/management involving commercial third-party wearable devices and genetic/biobank data; [45, 46]). It is important to highlight that many field-based athlete projects also feature individuals who are in “dual roles”—individuals that are actively on the research team and also provide athlete–practitioner services (e.g., nutrition or mental-performance advice; Table 2). These dual-role positions potentially feature existing relationships that require consideration of the power of relationship dynamics between practitioner and the research participant/athlete, which need to be carefully considered in the study protocol. One key step to minimize power of relationship dynamics is to ensure that all research consent is performed by someone exclusively on the research team with no prior relationship to the research participants.

**Table 2:** Ethics submission considerations unique to many field-based research projects

Ethical consideration	Potential mitigating solution/strategy
Athlete medical safety (physical/mental) during extreme competitions or training settings	Include appropriate medical and safety experts as part of the ethics submission, research protocol and event. For competitions, engage the event medical team, which can operate cooperatively—albeit independently—from the research team
Athlete confidentiality	By definition, there are very few world-class athletes [1] (i.e., world-record holders are singular), and by extension, many field-based studies have small participant numbers that can limit athlete confidentiality. Therefore, the consent process needs to highlight that full confidentiality might not always be possible. Nevertheless, the research project should include instructions for both athletes and research team members to avoid sharing information about other athletes without explicit permission (e.g., on social media or in presentations about the project)
Invasive procedures and athlete research burden	Research protocols like blood or muscle sampling, or the emotional burden of food tracking, can be especially challenging. Careful consideration and justification of every protocol and method must occur through the lens of a high-performance athlete. Research conducted at any competition must always prioritize performance versus research outcomes
Studies involving testing protocols or agents included on anti-doping lists	World-Class/Elite athletes should not be participants, as it will expose them to WADA anti-doping rule violations [2, 3]
Corporate sponsors or employed athletes associated with the event or the research	Informed consent must be provided without coercion or undue influence from the researchers, associated team coaches or contractual obligations (e.g., sponsorships). Ideally, the collection of informed consent should be undertaken by someone who is associated with the project, but not in a direct relationship with the athletes or the corporate sponsor. More specifically, athletes should be allowed to “opt-in” or “opt-out” of specific testing procedures/protocols within the research design, without personal ramifications (contractual or otherwise), and with ongoing consent re-evaluated throughout the research study. Where applicable, protocols should also be submitted to ClinicalTrials.gov to promote transparency and encourage the full disclosure of research outcomes
“Live” data generation and timing of athlete feedback	Many field-based studies implement direct monitoring that generates “live” data. However, “live” research data generated and disclosed to a athlete during a study (such as their pacing, core body temperature or fueling) could inadvertently alter athlete behavior, reducing the ecological validity of the outcomes. Similarly, ‘live’ research data could be used by the medical team to justify withdrawing an athlete from an event for health and safety reasons, with or without their consent. Therefore, as long as it is ethically sensible, we suggest providing data to athletes after study completion
“Dual roles”: Research staff who are also athlete support staff (e.g., sport science or medicine practitioners)	Individuals within the project team who are in a “dual role” acting both as a practitioner (e.g., nutrition, mental/physical performance) as well as researcher need to carefully manage ethical and study protocol considerations

Given the unique nature of many field-based elite athlete research projects, ethics approval can take weeks to months. In some instances, familiarizing an IRB board to field-based research can help establish methods and protocols that allow for a more streamlined process.

It should be noted that while field-based studies may not provide the highest quality of research design (many tend to implement cross-sectional designs) [47], these studies are often the only ethically-appropriate research designs to study scenarios involving competition outcomes, chronic disorders/injury research, and extreme training that many elite athletes undertake. For example, the only ethical way to assess the impact and outcomes of extreme conditions [e.g., such as athlete eating disorders, relative energy deficiency in sport (REDs) [48], or the extreme physical output required to train ~ 30,000 km/year and successfully finish the Tour de France [49, 50]] is to monitor cross-sectional outcomes in free-living athletes who have voluntarily chosen to undertake these activities. Although every IRB board is unique in its specific requirements, Table 2 highlights some common ethical considerations that are unique and beyond typical laboratory-based research.

### Logistical Considerations

A proactive approach to addressing logistical challenges is essential, as this allows the research team to fully focus on research activities and increases the likelihood of the highest quality data collection. Specific considerations and mitigation strategies are provided in Table 3. It is beneficial to organize a trial run of the event that includes representatives of all collaborating partners, both during the project development and immediately prior to the event. If an “in person” trial run is not possible, a virtual practice is recommended. We recommend including practical break-out/tabletop sessions to work through common field-based challenges and develop project-specific standard operating procedures [SOP; e.g., an athlete injury or a storm (for outdoor events), power outages, doping controls, medical emergencies, transportation logistics, etc.].

**Table 3:** Logistical considerations for the management of complex, multi-collaborator, field-based research projects

Logistical consideration	Potential mitigating solution/strategy
Ensure clear, regular and timely communication within the research team and with collaborators	Prior to the event Present an overview of all research elements to the entire research and event team, allowing space for questions and feedback Conduct regular meetings with frequent updates on relevant topics Proactively implement easily engageable rapid communication channels Identify specific personnel responsible for liaising with key collaborators (e.g., event organizers, corporate sponsors, athletes) At the event Hold daily team meetings to review and preview wins and challenges, while continuing to optimize and adjust plans and processes Schedule research team shift overlap to ensure handover of key information across teams
Establish clear roles and responsibilities across research team	Create an event organizational chart, identifying individuals responsible for a) the delivery of the overall project and b) specific sub-components (i.e., ‘sub-teams’ where required) Appoint project leads to liaise with collaborators, handle urgent challenges, and ultimately ensure sub-teams can focus on all research-specific activities For long duration field-based projects, implement a rotating schedule to provide the research team with adequate downtime Build redundancy within the research team to ensure data collection continues if a member is unable to perform their duties Where possible, ensure the research team has an extra member designated as a “runner” to provide all levels of logistical support
Research equipment considerations	Research equipment transportation/delivery (if required): mode, cost and speed of transportation (e.g., does equipment require special handling? How heavy and durable is the equipment? When does it need to arrive?) Customs permits and insurance needs (e.g., import/export licenses?) Equipment climate control requirements during shipping Security and storage (during shipping and the event) Power needs (e.g., sufficient power adaptors, cables, and plug sockets and is equipment compliant with the local power supply?)
Research and project infrastructure	If possible, the research team should be involved in designing onsite research infrastructure to provide efficient access to athletes and coaches, ensuring proximity, confidentiality, and minimal disruption and interference with performance needs Ideally, implement engagement with local experts (e.g., ‘champions’, ‘fixers’). Individuals with in-depth knowledge of the local area and community can assist with research demands (e.g., rental of key research instrumentation, contacts for local research collaborators) as well as general logistics (e.g., sourcing of general equipment and supplies)
Event Risk Management	Prior to the event prepare for emergency scenarios including research-specific risk planning and ensure that the resulting research program emergency action plan (EAP) has been shared with key collaborating partners, the research and the event medical team If possible, backup critical equipment and infrastructure (e.g., in case extreme weather or other factors damage essential equipment, lost luggage, etc.) and develop contingency plans for their quick replacement or repair Assign a research project lead to manage real-time emergency communication with key collaborating partners, to ensure critical updates are relayed to the team Ensure that all researchers are well-versed in the EAP and are aware of communication protocols, so they can act quickly and efficiently during a crisis
Trial run of the event	The methods / techniques of research can be unfamiliar to non-scientific collaborators. Therefore, provide a detailed walkthrough of the research plans, procedures, and expected outcomes. This will ensure that all personnel understand the infrastructure requirements and can contribute to effective and seamless workflow design Conducting a full-scale dry run of field testing using planned research equipment and data collection methods prior to the event is invaluable. This will provide clarity on how all aspects of the research will be conducted and coordinated, alleviating potential concerns among the research team and collaborating partners. Trial runs will identify unforeseen logistical or technical issues, allowing time for adjustments
Research team accommodation and transportation	Build in ‘buffer days’ at the field-testing location. This will allow researchers time to reschedule testing if unforeseen factors cause delays (e.g., weather/flight delays, cancellations, etc.) For longer duration events, researcher accommodation should be located as close as possible to the field-testing site minimizing travel time and fatigue If transportation is required, schedule assigned drivers and passengers for each vehicle to minimize complexity and ensure efficiency. Identify back-up drivers to account for any last-minute driver unavailability

### Corporate Involvement

Suggestions have been put forward to carefully manage corporate interests while ensuring scientific integrity in science [51, 52]. When appropriately managed, it is important to note that industry involvement does not always have negative consequences for science and can feature many benefits. Funding expansion is a clear benefit of corporate sponsorship, offering opportunities to increase the scale of field-based research and attract world-class athletes (and researchers) to participate. For instance, the Nike-sponsored Breaking2 project, which attempted to be the first to break the 2-h marathon barrier. While the goal was not achieved during the first attempt, the project generated extensive scientific discourse and contributed to the understanding of the limits of human performance [53, 54]. Further, Jones et al. published on the physiological demands of running a 2-h marathon [55], which has been cited 154 times during the first 3 years of publication and with findings contributing to a growing body of knowledge on fatigue resistance [56]. This is a great example of a corporate-sponsored event that simultaneously advanced scientific understanding.

In multi-collaborator projects, corporate partners may seek to enhance their brand image, researchers aim to collect high-quality data, and athletes want to showcase their talents and perform at their very best; achievement of these outcomes requires transparency, regular communication, and clear goal alignment. Given the various goals and the potential for miscommunication arising from differing business and scientific cultures, individuals who understand both environments can prove highly useful. These individuals can serve as bridges between collaborators, facilitating effective communication and cooperation and ensuring that the partnership advances both scientific goals and corporate objectives.

## Field-Based Physiological Methods and Data Collection Considerations

Monitoring an athlete’s physiological response to training and competition is fundamental to optimize training programs, recovery and health strategies, and overall athletic performance [57]. Physiological and performance testing in the field can primarily occur in three ways: (1) capturing data during verified competition/races; (2) creating scenarios that mimic competition environments; or (3) during training (in local environments or training camps). This may involve a mixture of noninvasive methods, use of portable or wearable devices, and/or the creation of “make-shift” laboratories at an athlete’s training/race location. More recently, the term “invisible monitoring” has emerged, which has recently been defined as “a set of techniques and associated data analysis allowing the measurement of a single or multiple training effects using a single or combination of measurement tools with a minimal degree of burden to athlete and staff” [58]. Leduc and Weaving have recently reviewed the opportunities and challenges with invisible monitoring in field-based situations [58], much of which is expanded below.

### Wearable and Portable Measurement Devices

Emerging wearable devices may enhance the quantity and data collection frequency; however, the use requires consideration regarding device accuracy, reliability and validity, and data privacy (see Sect. 4: Wearables for more information [59, 60]). Physiological variables for the quantification of training zones or adaptive responses can now be measured using portable devices. Technological advancements in recent decades have meant that key variables such as heart rate, power output, speed, core temperature, and movement patterns can all be measured continuously during races or in sport contexts. Devices such as smart watches, heart rate straps, GPS units, and power meters allow for data to be either stored or transmitted in real-time. Other physiological tests that can easily be conducted in the field include the assessment of sweat rates, determination of perceived psychological states (i.e., rating of perceived exertion scales), and measurements of blood metabolites. In particular, many blood markers (e.g., lactate) can now be assessed rapidly, and with minimal sample volumes, using portable analyzers (sometimes in < 15 s with just ~ 0.3 µl of the sample).

Emerging technologies have allowed for the development of portable indirect calorimeters, but several factors need to be considered prior to implementation. Equipment should be durable to withstand potentially harsh transit and field-based conditions. It is also necessary to know the equipment measurement precision and variability, as well as the sources of technical and biological measurement variability—as various indirect portable and/or lab-based indirect calorimeters can both under- and overestimate volume of oxygen (VO_2_; range: − 8 to + 18%) [61, 62] and may have reduced accuracy when ventilation is high, when deriving substrate oxidation rates or when analyzers do not correct for gas temperature. Sources of error should be minimized, standardized, and accounted for in the interpretation of results [63]. Although many indirect calorimeters account for environmental factors (i.e., temperature, pressure, etc.) during calibration, not all devices have this capability, potentially affecting accuracy in extreme conditions (e.g., high elevation). Where possible, maintaining an ambient temperature between 20 and 25 °C during testing is important to maximize accuracy [63, 64]. In addition, some portable indirect calorimeters (e.g., Cosmed K5) can accurately determine gas volumes and EEE at lower exercise intensities [62, 65, 66], but not all devices are accurate at very low (i.e., resting energy expenditure) or very high (i.e., high-intensity exercise) detection limits.

Portable near-infrared spectroscopy (NIRS) noninvasively measures muscle oxygenation levels and vascular function by attaching a small, lightweight device over the muscle of interest. It has been shown that NIRS is valid for measuring local oxygen saturation (SmO_2_) but not total hemoglobin [67]. As exercise intensity increases, reliability issues arise, possibly owing to tissue ischemia or an increase in movement artifacts associated with higher frequency of muscle contraction [68, 69].

In summary, it is important to understand the typical error of measurement/variability for both the equipment and the primary variable(s) in the proposed field-based setting. Many portable field-based technologies tend to not only have higher measurement error and/or variability than laboratory methods but also the potential for greater data sampling, which potentially can reduce sampling bias and improve reliability. Nevertheless, the implementation of small, portable technologies allows for field-based data capture to occur in locations unique to sport (e.g., poolside, from a traveling automobile, or in environmentally challenging terrain; a comprehensive list is provided in Supplementary Table 2).

### Field-Based Laboratory Assembly

The establishment of field-based laboratories allows for more sophisticated measures of physiological metrics. There are numerous examples in literature where this has been successfully implemented in the elite athlete population [18, 70, 71]. However, to obtain valid and reliable field-based data, many logistics require consideration. On the basis of the authors’ experiences, logistics applicable to most research questions are outlined in Table 4.

**Table 4:** General logistical considerations for assembling field-based physiological testing spaces for research purposes with athletes

Environmental conditions	Thorough data capture of environmental conditions allows a greater understanding of data anomalies and data interpretation **Outdoor testing** If rain/snow/wind is expected, ensure adequate waterproofing of equipment. Ensure no safety hazards (e.g., slips) arise from wet floors and/or equipment If in hot/sunny conditions, provide adequate sun protection and cooling measures (within scope of study design) Measure and record environmental conditions (temperature, humidity, barometric pressure, and wind speed) systematically throughout the entire testing period or regularly throughout training and/or competition Understand the functioning temperature range of your equipment, especially when working in extreme environmental conditions **Indoor testing** Unless extreme temperatures are required for the research protocol, aim to keep environments as thermoneutral as possible. Temperatures of between 18–23 degrees and relative humidity < 70% are recommended [1] not only for athlete comfort, but for reliable and accurate testing results. It may be necessary to source portable heating/cooling systems When the measurement of gases and expired air is required, consider and control the effect of temperature on gas volume, pressure and density As the thermal environment is difficult to control, measure environmental conditions frequently to allow for early intervention
Lighting	Check facility lighting prior to use. If testing in the early morning, or late evening, good lighting is critical. Importantly, this may not be obvious during the day Determine the facilities capacity for lighting adjustments as required (e.g., dark rooms for RMR measurements) Consider additional equipment such as headlamps and batteries (e.g., for performing venipunctures/cannulation/RMR in poorly lit rooms), if required
Power sources	A stable power supply can be a challenge in the field, particularly with expensive research-grade equipment. Generators are not always safe or reliable and can produce inconsistent power surges. Surge protectors and a battery tower may be necessary If bringing equipment into a new testing location, pre-check the voltage requirements and power sockets. Larger items, such as metabolic carts or treadmills may have special requirements. These should be checked prior to testing to avoid inadvertent outages due to circuit overload If traveling internationally with equipment, confirm that adaptors can handle the required voltage and that any required importation paperwork is in order Where required, liaise with the building manager or event organizer in advance to fully understand the electric/power provision and options (e.g., use of generators) Always travel with additional power adaptors and extension cords Depending on study requirements, a portable external battery may provide peace of mind
Calibration	Always complete a calibration check on equipment that has been moved If calibration requires the use of specific gases or reagents, these should travel with the equipment Be mindful of the transportation and storage requirements for compressed (e.g., calibration gases, medical grade oxygen) and dangerous gases (carbon monoxide). These are likely to differ between states and countries
Cleaning and biohazard considerations	Designate ample space for equipment cleaning. Specific protocols should be created for each testing facility to ensure that laboratory SOP cleaning standards are met Ensure sufficient cleaning products, tools and drying racks are available. Non-researchers may be present in the lab space; label cleaning products and spaces accordingly Some equipment requires higher cleaning standards (e.g., mouthpieces) or specialized cleaning procedures. Designate space for these activities Ensure that adequate biohazard bins and sharps containers are available if working with biohazardous material. A waste disposal plan must also be created that complies with local laws and procedures
Health and safety	Staff wellbeing during long testing days in competition/camp-based scenarios is fundamental. Ensuring adequate rest/sleep and access to good nutrition will allow staff to manage multiple testing days and maximize the likelihood that data capture occurs without errors or mistakes Staff should maintain usual laboratory dress codes (e.g., enclosed shoes, hair tied back) Ensure a well-stocked first aid kit is rapidly available if required. Key staff should also hold current CPR and first aid accreditations Develop an EAP specific to your testing location(s). At a minimum, all staff should be clearly aware of the local emergency phone number and the nearest automated external defibrillator and hospital locations Make sure the space is well stocked with items such as hand sanitizer, gloves, hand wash, disinfectant wipes If chemicals or dangerous gases are required in the testing space (D2O, glycerol, carbon monoxide) ensure items are clearly labeled, and specific safety processes are followed
Sound	Where appropriate, ensure a portable sound system is available so that music can be played for entertainment or to encourage peak performance (e.g., VO_2_max test) For testing that requires silence (e.g., RMR assessments, cognitive tests), minimize noise from surrounding individuals and/or the environment. If not possible, provide ear plugs and eye masks to increase test validity
Transport	Depending on the location of the athlete’s accommodation and training facilities, travel between sites may be required. Consideration should be given to the number of cars (and drivers with appropriate licenses) and/or bicycles that may be needed

CPR, cardiopulmonary resuscitation; D2O, deuterium labeled water (heavy water); EAP, emergency action plan; RMR, resting metabolic rate; SOP, standard operation procedure

### Research Testing Scheduling and Testing Burden Considerations for Elite Athletes

The scheduling of research testing protocols must consider the athlete’s training program and available free time. Testing schedules that significantly interfere with an athlete’s training or represent a significant burden (e.g., tests to exhaustion or prolonged fasting) are likely to be unfavorably regarded by coaches and athletes and may lead to poor athlete recruitment or compliance. To address recruitment and athlete compliance challenges, high-impact protocols that provide immediate and noticeable benefits to the athlete and coaches should be prioritized. The cost of implementation (both financial and time) and invasiveness of the protocol also factor into the decision-making process. One approach to reducing the time burden of most tests is to increase the available staff, equipment, and resources, ensuring careful choreography of schedules to enable a higher throughput of athletes. For example, with one metabolic cart and two staff members, a graded exercise test (GXT) may “cost” 60 min/athlete (inclusive of preparation, recalibration, and cleaning). However, with multiple metabolic carts and ample staff members, GXT efficiency may increase to 20 min/athlete. In addition, arranging phlebotomists to undertake blood draws directly at the athlete’s accommodations/training locations, rather than having athletes travel back and forth to a laboratory, can be a significant time-saver. Finally, it is important to consider how and when testing protocols will be integrated into an athlete’s training plan. For example, scheduling a maximal GXT on an athlete’s rest day is likely to negatively impact load management and recovery and, possibly, an athlete’s willingness to comply. Other scheduling considerations include the required nutritional status (fasted versus fed), timing of the last exercise session, and use of ergogenic aids. In instances where repeated testing is involved, standardization and/or replication of equipment, nutritional status (where relevant), and time of testing should occur. A research program co-designed with input from the athlete and coach is likely to yield the most successful results.

## Field-Based Wearable Considerations

Wearables, defined as “body-borne computational and sensory devices which can sense the person who wears them and/or their environment” [72] have advanced significantly in both technology and widespread adoption [39]—ranking as the largest fitness trend in 2024 [73]. In sport, wearable technology has become a mainstay, with training and competition data used to improve personalized training prescription, to enhance competition strategy, and to better quantify training or competition loads and recovery requirements [74]. The enhanced technological capacities and improved usability have made wearable technology highly relevant to field-based sport research and accelerated the number of publications featuring wearable technology data.

Wearable technologies allow real-time monitoring of physiological, biomechanical, and environmental data with high sampling frequencies and resolution [74]. Parameters such as blood glucose levels and running gait biomechanics can now be continuously monitored during field-based events. This enhances ecological validity and offers specific insights into the demands of the sport, improving our understanding of world-class performance. Modern wearable sensors are lightweight and unobtrusive. Hence, athletes and coaches are more likely to agree to their use. However, given the rapid advances and relative novelty of wearable sensor use in research, challenges remain in the validity and applicability of results [24].

**Fig. 2:**
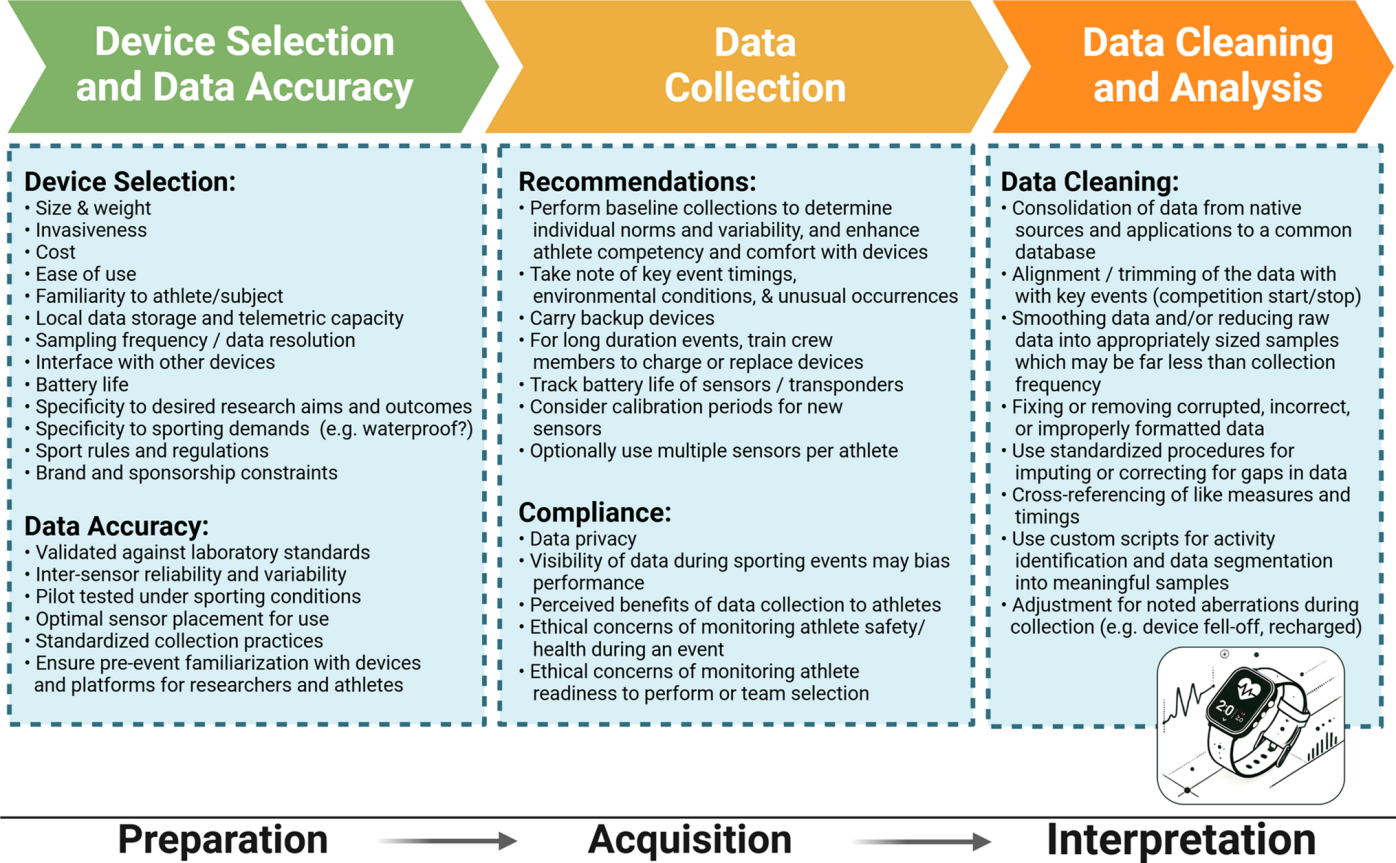
Technical considerations for the use of wearable technology in field-based research

When planning, collecting, and interpreting field-based wearables data, several factors must be carefully considered to ensure data integrity and minimize bias (Fig. 2). During the study design phase, crucial considerations include the ethics of wearable use, as well as selecting devices with resolution and accuracy that align with the study’s objectives and the sport. Devices must comply with the rules of competition. For example, many sports do not allow information transmission to athletes during competitions; some sports have strict regulations around data collection and storage, while others restrict the equipment that can be worn during competition (e.g., watches, jewelry, etc.). These restrictions are particularly true in professional sport where collective bargaining agreements can define what, when, and how data can be used with athletes [75]. Researchers and athletes need to carefully consider who will have access to the data and whether coaches or persons in power will be able to make real-time decisions that could influence performance and safety. The potential for data to bias athletic performance and the resultant implications regarding athlete decisions (i.e., playing time, injury management) are critical factors requiring careful deliberation. Many wearables also feature third-party commercialized “apps” that require critical consideration regarding who has information access and subject confidentiality (which is especially relevant in IRB ethics applications; see Sect. 2.2.2/Table 2). This is especially true of the plethora of period/menstrual tracking apps [76]. For example, many menstrual apps may “claim” privacy, but some apps still sell or use collected personal health data, which may have considerable potential ramifications in the USA in the post *Roe v. Wade* era [76, 77]. Unfortunately, there are no global ethical or best-practice standards for the use of wearables in sports [60].

Effective data collection requires standardized procedures to reduce variability, while also accounting for device limitations and both expected and unexpected deviations in collection protocols. For instance, in individual endurance events, specific notation of key events during data collection (e.g., event start/finish, nutrition timing, rest intervals) can strongly support subsequent analysis. In scenarios where the test cannot be repeated (e.g., during competition), it is prudent to prepare for potential failures with spare batteries, devices, and duplicate measures, when possible. Conversely, in team sports, time-outs, substitutions, player changes, and practice stops/downtime also require specific notation and provide challenges for later analysis [78, 79]. A recent report highlights a multidiscipline three-element approach of “minimal, adequate, and accurate” sport science support in real-world conditions. This study showcases positive aspects of strategically implementing mainly wearable and noninvasive monitoring that satisfies the minimal, adequate, and accurate elements, allowing for enhanced, informed decision-making with world-class sprinters [80].

Interpreting the data collected from wearables also poses unique challenges. Careful systematic consideration must be given to the context in which the data were collected to draw valid and applicable conclusions. This includes factors such as potential biases/variability introduced by device inaccuracies, environmental factors, and variability between and within athletes. By meticulously planning the selection of devices, standardizing data collection protocols, and thoroughly considering the context and potential biases during data interpretation, researchers can improve the robustness of their results.

## Field-Based Motion-Capture Considerations

Technological advances have made it feasible to capture high-quality field-based biomechanical data without the use of reflective markers. Markerless motion capture (MMC) provides high-fidelity data in complex or restrictive environments using advanced computer vision and machine learning algorithms to track joint movements directly from high-resolution video footage [81, 82]. This allows researchers to study athletes in their natural environments, with minimal disruption. Although MMC expands the scope and ecological validity of biomechanical research into field-based settings, there are several logistical and technical challenges that must be addressed to ensure accurate and reliable data capture.

### MMC System, Cameras, and Data Management

MMC system selection is guided by factors such as activity type, environment, and the desired biomechanical outcomes. Systems such as Theia3D or Vicon may offer superior accuracy, while the open-source platform OpenCap provides better accessibility and price but lower accuracy [83, 84]. Proper camera selection and configuration involves: (a) optimal placement of cameras to ensure overlapping fields of view, necessary for accurate three-dimensional reconstruction of movements; (b) implementing higher frame rates for capturing fast movements; (c) higher resolutions to improve the accuracy of landmark detection [85] and; (d) optimizing exposure settings to ensure a balance between brightness and clarity [86, 87]. MMC systems generate vast amounts of data, especially with multiple testing sessions over days (> 1 terabyte of data), requiring robust processing and storage solutions with substantial computational power. Onboard storage options in some cameras can offload processing demands from computers and provide redundancy in cases of data loss. To provide a more comprehensive biomechanical field-based assessment, MMC data can be integrated with other biomechanical sensors such as force plates and inertial measurement units (IMUs; see Wearables, Sect. 4) [88, 89]. However, multi-sensor integration requires careful synchronization and calibration to ensure that all data sources align accurately [84, 88].

### Lighting and Environmental Conditions

Proper lighting is one of the most critical factors influencing the quality of MMC data, with inconsistent or low lighting impairing anatomical landmark detection accuracy [90]. For field-based assessment, controlled lighting setups, such as opaque tents and artificial lighting at night, are strongly recommended. Field-based research often involves varying and extreme weather conditions (e.g., wind, rain, and heat), which can affect camera performance and equipment functionality. Adverse environmental conditions can be circumvented by stabilizing tripods and adequately protecting equipment using a durable tent with robust sides. Frequent calibration and continuously optimizing camera settings is critical to maintain high MMC reliability [85, 86, 91, 92]. While intra-session reliability is generally high, inter-session reliability can vary, particularly with changes in setup or conditions [91, 93].

### Sport-Specific Considerations

MMC is particularly suited for capturing large, planar movements in sagittal views (e.g., walking/running) [84, 85, 94, 95]. Conversely, MMC lags behind traditional markered systems for capturing complex joint angles and movements involving smaller body segments (e.g., intricate hand movements) and frontal views, which require careful calibration for accuracy [83, 85, 89, 95]. When simultaneously tracking multiple athletes, additional challenges arise, such as ensuring accurate identification and individualized tracking. Reliability can also vary with different sports and movement types, generally being higher in linear, repetitive movements, such as running, and lower in more variable, complex, multi-planar activities [91, 92].

In summary, we strongly recommend piloting all systems/methods prior to actual data collection. With meticulous planning, researchers can improve the reliability and applicability of the data collected. Accordingly, MMC can be a powerful tool for enhancing the understanding of athletic performance, ultimately contributing deeper insights into how athletes perform in their natural field-based environment.

## Considerations for Field-Based Assessment of Neuromuscular and Cognitive-Motor Fatigue

In studies of neuromuscular or cognitive-motor function, researchers aim to control many factors that could influence the data, such as time of day [96, 97], caffeine [98], sleep [99], and, potentially, menstrual cycle phase [100]. Although field-based testing with athletes can make it challenging to control for all these variables, they should be considered when possible and acknowledged when not.

Power is the central neuromuscular assessment associated with dynamic sport activities. However, even in a laboratory setting, equipment limitations (e.g., cost of an isokinetic dynamometer and restricted relevance of constant-velocity contractions) mean that muscle force or torque is the most commonly measured variable. In field-based studies, it is necessary to use rigid materials that can also be easily disassembled for transport to field-based locations, such as 80:20 aluminum framing, for myograph design for optimal stability to accurately measure maximal force/torque.

Measurement of maximal force/torque before and after training/competition enables quantification of exercise-induced impairments to muscle capacity (i.e., neuromuscular fatigue) but does not offer insight into the mechanisms responsible for the fatigue. To achieve these insights, external stimulation is required to evaluate the intrinsic contractile capacity of the muscle as well as the effectiveness of the central nervous system (CNS) in activating the muscle [101]. Unfortunately, some athletes opt out of testing owing to unfamiliarity with stimulation, the associated transient discomfort, and the perception that this might negatively impact performance. It is important, therefore, to consider the form of stimulation needed to obtain the desired physiological data; this must be weighed against the discomfort it causes. To evaluate voluntary activation (completeness of drive from the CNS during a maximal voluntary contraction [102]), it is appropriate to use easily tolerated single stimuli [103]. However, if the focus is intrinsic contractile function, the physiological relevance of the force/torque response to a single stimulus when the muscle is relaxed is lower than the response to trains of repeated stimuli. This can cause more discomfort but more closely mimics how the CNS activates muscles during voluntary contractions. Regardless, to increase athlete buy-in, it is critical to underscore the value of the information that will be obtained and, ideally, familiarize them with stimulation before actual data collection and to educate and/or demonstrate that the tests do not negatively impact performance.

As with neuromuscular assessment, the need for field-based portability at athlete training and competition locations will impose limitations and exclude cutting-edge laboratory-based technologies (e.g., KINARM end-point robot; [104]). Fortunately, there are numerous tablet/laptop-based options available (e.g., INPL Trail Making Test; Rabien Software or Presentation; Neurobehavioral Systems, Berkeley, USA) that allow a comprehensive assessment.

Cognitive-motor tests can require many practice trials to attenuate learning effects [105]. Therefore, to optimize accurate measurement of training/competition-induced impairments, researchers must provide athletes with sufficient practice before actual testing. For some athletes, this commitment can be off-putting; therefore, the duration and timing of testing requires careful consideration. In addition, cognition-motor testing should take place in a distraction-free space, which can be difficult to arrange during field-based testing. Ideally, researchers should also implement over-ear noise-canceling headphones with white noise to limit the influence of environmental noise. Taken together, with the development of sturdy, yet portable, myographs, and the proliferation of various tablet based cognitive performance tools, the assessment of neuromuscular and cognitive-motor fatigue can be a consideration in any athlete field-based study.

## Field-Based Psychosocial Considerations

In psychosocial sport research, field-based methods are integral to understanding how personal and environmental factors interact to shape athletes’ experiences. Field-based methods typically include both quantitative and qualitative approaches to gain insight into athletes’ psychological processes (thoughts, emotions, and behaviors). Valid and rigorous psychosocial research considers the highly complex and dynamic nature of the varied personal and environmental factors that explain psychological phenomena [106]. Quantitative methods are often used to identify and explain relationships among constructs (i.e., psychological phenomena that cannot be seen objectively) with large sport populations (e.g., 100–800 athletes). For psychological phenomena that involve targeted samples (e.g., all elite sport samples), qualitative exploratory research is useful in unearthing potential mediators and moderators and extraneous variables that shape how athletes navigate their sporting environments in the pursuit of optimal performance [107].

As an example, our research team recently sought to identify and understand the psychosocial experiences of women athletes in preparation for a field-based 6-day ultra-distance race. Specifically, the constructs of self-regulation, pain, body image, and gender were pre-identified for study. We employed a constructivist qualitative research design that included researcher observations [108] and semistructured interviews [109] at multiple time points to capture the complex and dynamic psychosocial experience of interest. This method permitted a centering of the perspectives of the women themselves by speaking to them about their experiences. The longitudinal design allowed time to develop rapport and to deeply embed ourselves (i.e., the researchers) within their training and competitive and personal environments [110]. In a series of three qualitative semistructured interviews over a 7-month period, we asked athletes to share their experiences in training and competition. The goal was to interpret the meanings they assigned to their experiences leading up to, during, and following a targeted competitive event. We also conducted researcher observations at a training camp and at the competition itself, allowing us to observe athletes in real-time to learn about the multifarious contextual and personal factors that interacted to shape how they navigated their training and performance contexts in situ. These direct field-based observations also contextualized what the women had shared in interviews, catalyzing a more nuanced understanding of their emotional, cognitive, and behavioral responses in and outside of training and competition.

A constructivist qualitative design presents a massive breadth of potential data that can be gleaned during interviews and observations. This presents a challenge when aiming to generate, analyze, and interpret qualitative data. Important decisions need to be made by researchers, in collaboration with athletes themselves, on which psychosocial aspects are most important for the research question. In the example presented, pre-competition interview data can be helpful in guiding the identification of key observational goals. In addition, post-competition interviews with athletes garnered their perspectives on researcher interpretations, which, in turn, acted as a member reflection, contributing to methodological rigor [109].

Elite athletes are routinely subjected to highly stressful sport environments and most have fully developed psychological competencies required to adaptively navigate these challenges [111]. Learning from athletes themselves through situations of high ecological validity in the field, through qualitative interviews and observations, allows researchers and practitioners to devise interventions that better meet athletes’ needs and that consider important contextual factors (e.g., gender, race event logistics, organizational demands). The addition of psychosocial research approaches is often overlooked in sport field-based research projects. The systematic description of lived-experiences within the context of field-based research can be invaluable in concert with quantitative data.

## Field-Based Energetic Assessment Considerations

**Fig. 3:**
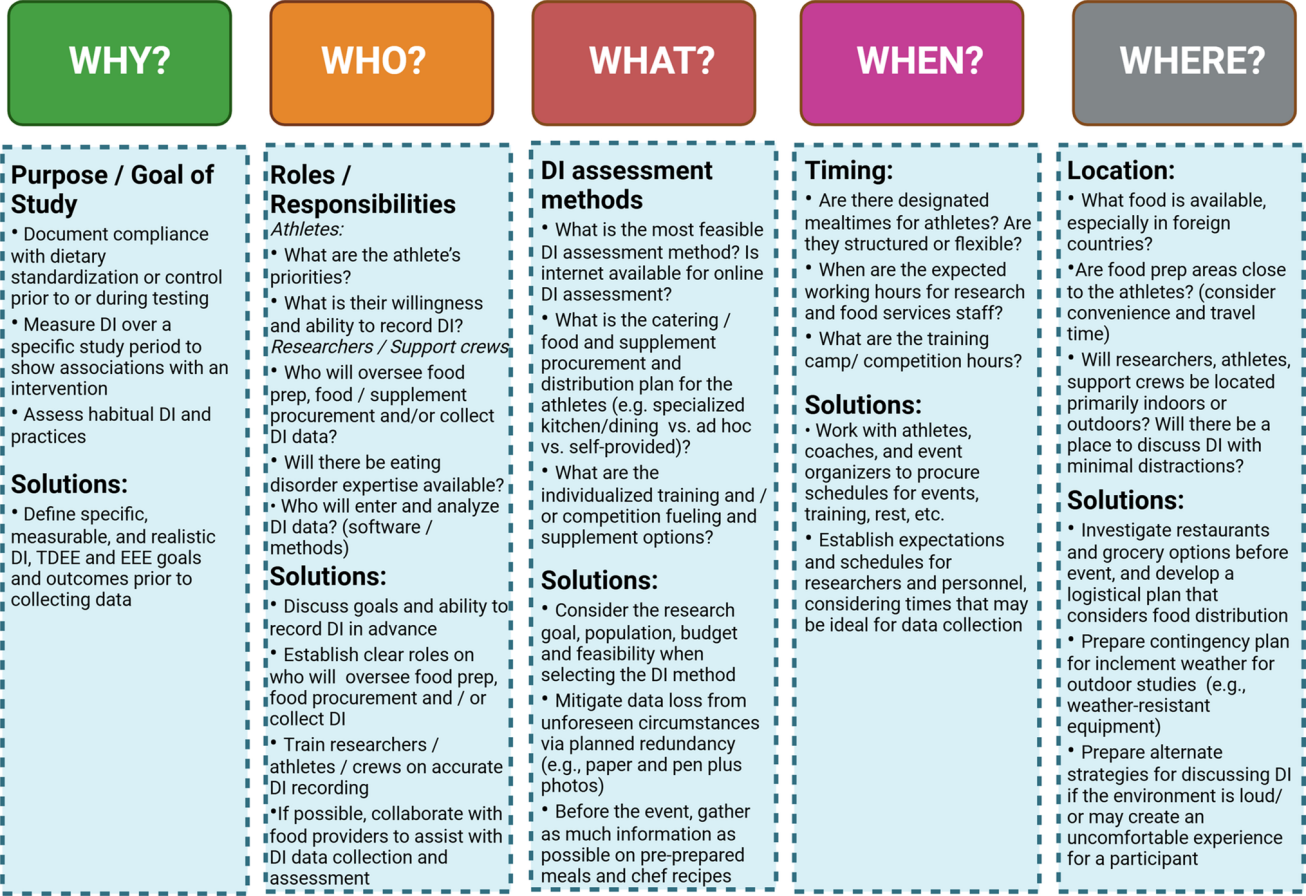
Logistical considerations for the measurement of dietary intake in field-based human performance research. *DI* dietary intake

The control and/or measurement of dietary intake (DI) includes capturing specific aspects of diet such as energy intake [EI; “calories” (kcal) consumed], micro- or macronutrients, phytonutrients, and water intake. It may also encompass factors such as dietary patterns, meal timing, or eating behaviors (Fig. 3). DI, along with resting energy expenditure (REE) and total daily energy expenditure (TDEE)—which includes EEE—are critical methodological components of many field-based athlete research studies. Owing to the often extreme nature of training loads or competition durations, the collection and measurement of EI, EEE, and TDEE in athletic populations can present significant challenges. In particular, researchers need to determine whether to control or assess DI and EEE for a specific time frame or to measure habitual patterns. The direct or indirect effects of assessment protocols on eating or exercise patterns also need consideration as they may impact habitual practices. In addition, researchers must understand behavioral influences or antecedents [112, 113] to avoid unintentionally impacting athlete behavior in the research environment (Fig. 3). Other publications have focused on dietary standardization strategies during performance testing in athletes [114] and dietary interventions in a camp-based setting [19]. Therefore, this section will focus on DI, TDEE, EEE, and REE in field-based scenarios.

### Dietary Intake Quantification

The context and goals of field-based research will inform the choice of DI data collection method, as each approach has various logistical and compliance considerations (Fig. 1). Regardless of the method, documenting adherence to DI standardization protocols is critical [114]. A DI record can establish if the athlete has met study requirements, such as following their usual routine, abstaining from a dietary component (e.g., caffeine or alcohol) in the day(s) leading up to testing, meeting a specific fueling target, or ensuring DI replication on further testing occasions [114]. In other scenarios, data may be used to investigate the effects of an intervention on DI [115] or the effects of DI on the intervention [116]. Other field-based research may include documenting athletes’ habitual dietary patterns [117–120] or their dietary patterns during prolonged competition [50, 121]. These data can then be compared with sports nutrition guidelines, changes in intakes over time, or associations with a metric of interest (e.g., performance).

Capturing accurate DI data is challenging—even with technological advances [122, 123]. The process can be burdensome for both researchers and athletes. It is also affected by errors in food and beverage quantification and identification, incomplete or erroneous food composition data, and the failure of the capture period to fully represent true DI [124, 125]. Further, data errors and the burden may also be affected by factors related to the athletic event environment, such as increased DI frequency and quantity, consumption of sports foods and supplements, and the inconsistency or impracticality of recording DI with a busy lifestyle (e.g., during exercise, while traveling) [126, 127]. Indeed, a review of available literature comparing self-reported EI with objective assessments of TDEE in athletes demonstrated a mean underestimation of 19% and considerable variability in the magnitude and direction of error between individuals (− 2793 ± 1134 kJ/day) [128]. Furthermore, the day to day variability of EI and EEE, representing energy availability, in free-living athletes is not always reflected in more strictly controlled laboratory-based research [129]. Researcher experience and athlete training in DI assessment prior to data collection can enhance accuracy and feasibility [124]. Ideally, researchers will also have ongoing contact and communication throughout the study period to establish and maintain trust to further optimize accuracy.

Each method for DI assessment has strengths and drawbacks [122], which are summarized in Fig. 3 and Table 5. Balancing feasibility and accuracy with financial expenses is crucial, particularly given the high cost of approaches such as weighed records via food scales or measurement of biomarkers. Equipment and tools must be portable, durable, and (often) suitable for outdoor use. Researchers must also account for the time, training, and effort required for recording food and beverages. Contingency plans and logistical planning that consider the goal of DI assessment, the athlete, the researcher expertise, and the environment (e.g., physical layout, schedule) can help optimize the data collection process. Furthermore, aspects of DI tracking may sometimes exacerbate disordered eating/eating disorders in some athletes [130]; therefore, researchers should be especially mindful in their research protocols, screening of athletes, and continued vigilance throughout research protocols to ensure no harm. It may be appropriate to seek advice from a professional with expertise in eating disorder management to assist with the design and implementation of DI tracking protocols [131]. Researchers must also consider data collection timing. Ideally, this occurs rapidly in field-based settings to support ecological validity without interfering with athletes’ schedules. This approach also circumvents long intervals between data recording and analysis, which has been shown to reduce accuracy [132, 133]. Adapting assessments to athletes’ preferences, readiness, schedules, and routines can improve adherence and minimize disruption.

**Table 5:** Considerations for selecting a dietary intake quantification tool in field-based human performance research

Retrospective			Prospective			
	Food frequency questionnaire (FFQs)	24 h recall	Food records	Food photography/phone applications	Direct observation/weigh and measure	Biomarkers
Accuracy for dietary intake in individuals	Low; designed to capture trends in groups, over long periods of time	Low; captures a single day, relies on memory and honesty	Moderate; can be accurate with multiple days and athlete’s engagement	Moderate; can be accurate if used consistently and correctly, with researcher oversight	High	Very high; for intake of energy or a specific nutrient
Equipment requirements	Low; paper/pen or computer	Low; paper/pen or computer	Low; paper/pen or computer	Low to moderate; smartphone, camera and/or app; may need internet access; may need pen/paper or text to clarify food image if not using an app	Moderate; food scales, measuring tools, system for recording data (paper/pen, software)	Very high; biological sample collection materials, often refrigerator or freezer for temporary storage, specialized laboratory equipment for analyses
Athlete commitment	Low to moderate; depending on FFQ length, but may exacerbate ED thoughts, feelings, or behaviors	Low to moderate; depending on the number of 24 h recalls, but may exacerbate ED thoughts, feelings, or behaviors	High; athletes record everything consumed over a specified time-frame (usually 3–7 days); may exacerbate ED thoughts, feelings, or behaviors	Moderate; athletes record everything consumed, but use of photos/apps can be more convenient than paper; may exacerbate ED thoughts, feelings, or behaviors	Low to moderate; athletes only need to provide opportunity for food review before and after consumption; may exacerbate ED thoughts, feelings, or behaviors	Low to moderate; athletes need to provide biological sample(s), which can be invasive or difficult in the field
Researcher training and time commitment	Low to moderate; depending on data entry and analysis; some automated FFQs are available	Moderate; requires trained interviewer to collect, enter, and analyze data; some automated 24h recalls are available	High; requires trained interviewer to collect, enter, and analyze data; data entry is time consuming	Moderate to high; requires trained personnel/ researchers to collect, enter, and analyze data; may require special software and nutrition-specific knowledge to analyze data	Very high; requires substantial time and researchers trained in weighing food and entering and analyzing data	Very high; requires several highly trained researchers for sample collection and analyses
Cost	Low, especially if intake entry and analyses are automated	Low, especially if intake entry and analyses are automatedLow, especially if intake entry and analyses are automated	Moderate, considering the need for trained personnel/ researchers and data entry	Low to moderate, depending on research team experience, app, or software	High, considering costs of personnel/ researcher labor	Very high, considering equipment, personnel/ researcher labor, and analyses

“Phone application” includes applications such as MyFitnessPal or Chronometer

ED, eating disorder

Understanding the geographical location, event layout, and the logistics of food provision is critical as disruptions to food availability could negatively impact performance, athlete well-being, and study compliance. Food availability challenges may inadvertently redirect researcher attention toward food provision logistics, rather than research, particularly when events occur in regions where the available foods or catering arrangements differ from the athletes’ usual diet and eating patterns. Thus, pre-camp planning should include communicating with the site and athletes to determine if suitable and familiar food options will be available in appropriate amounts and time periods, particularly as it relates to international travel and international athletes. Proactively assigning one researcher (or chef) to manage food logistics during the project will allow the remaining team to support research-specific DI assessment.

### Total Daily Energy Expenditure

TDEE consists of REE, activity energy expenditure, and the thermic effect of food (usually assumed to be 10% of TDEE) [134, 135]. Activity energy expenditure is divided into EEE [i.e., exercise energy expenditure from activity (training or competition) that is planned and structured] and non-exercise activity thermogenesis (NEAT; energy expenditure from all remaining activities such as work, leisure, household tasks, transportation, fidgeting, and body posture) [134, 135]. As previously reviewed [134–137], there are several approaches for measuring each component of TDEE. While many studies use wearable technology to estimate EEE and/or NEAT, most devices underestimate energy expenditure, particularly in athletes [138–141]. For brevity, the following discussion focuses on considerations for measuring TDEE and EEE using the most common field-based indirect calorimetry approaches [i.e., portable indirect calorimeters and doubly labeled water (DLW; ^2^H_2_^18^O)].

#### REE and EEE Measurement Considerations

There are several logistical considerations for measuring REE and EEE, using either portable indirect calorimeters, including pre-test protocols, sex hormone status, training location, and lifestyle. To increase REE measurement accuracy, athletes are recommended to fast for a minimum of 5 h and refrain from excessive exercise 14 h prior to testing [63]. However, these recommendations have been primarily developed in sedentary to recreational athletes and may not be valid with elite athletes. In addition, some research suggests that menstrual cycles impact REE [142]. However, more recent research suggests that menstrual cycles and hormonal contraceptives have minimal impact on REE in athletes [143], perhaps owing to greater overall energy expenditure in this population. As these data are mixed, hormonal contraceptive status and, ideally, sex hormone concentrations, should be documented in relation to energy balance until further athlete research is conducted. Regarding training location and lifestyle, some studies have shown minimal or limited effects of altitude [144] or of diet and exercise manipulation the day before REE measurement [145] in athletes. Conversely, diet and exercise can have profound acute effects (up to ~ 2–3% changes) on fat-free mass assessment via dual X-ray absorptiometry (DXA) in athletes [145–147]. Given these data are typically used in REE assessments, diet and exercise should be recorded and potentially controlled in the 24-h period prior to DXA scans. Finally, sleep, stress, and altered circadian rhythms (if traveling) may impact REE [148–150]. Careful scheduling of REE is, therefore, crucial to minimize both measurement variability and disruption to an athlete’s routine. Portable calorimeters that measure both oxygen and carbon dioxide offer greater REE assessment accuracy [151, 152] than devices that only measure the concentration and volume of a single gas [153–155]. Further extensive discussion on the selection and consideration of portable indirect calorimetry has been mentioned above in Sect. 3: Physiological Considerations and Table 4.

#### TDEE: Doubly Labeled Water

DLW is ideal for measuring TDEE in field-based settings, as it is relatively noninvasive and robust to different sampling and storage environments [156] (Table 6). DLW involves the collection of a pre-dose urine sample, followed by consumption of a precisely weighed dose of ^2^H_2_^18^O labeled water. Additional urine samples are collected in the following 3–10 h to determine DLW peak equilibration within the body. Depending on duration of the protocol, and the desired time-course of sampling, only a few urine samples are required over the next 7–14 days. However, it can sometimes be logistically challenging to coordinate sample collection and to ensure that athletes can provide multiple samples on dosing day. Researchers should implement multiple methods for recording DLW dose, athlete body mass, and timing to avoid potential data loss. DLW isotopes are resistant to breakdown, thus, sample refrigeration or freezing is not required but will minimize bacterial growth and odors. As biological samples are susceptible to evaporation, which can impact isotope concentration, they should immediately be sealed with air-tight caps. Samples must be shipped to an analytical laboratory, requiring knowledge of transport requirements for biological materials. It is useful to retain backup samples until safe receipt of samples and successful analysis at the analytical laboratory are confirmed.

**Table 6:** Key considerations of measuring resting energy expenditure (REE) and total daily energy expenditure (TDEE) in field-based human performance research

REE	
Athletes	Ability to adhere to pre-test protocols (e.g., fasting, avoiding vigorous exercise) Schedule to minimize interference with down time, training sessions or competitions Sleep, stress, and altered circadian rhythms (if jet lagged from traveling) may impact measurements
Researcher	Experience (and/or training) in proper use/maintenance of equipment Availability aligns with athlete schedules
Equipment	Ensuring equipment can withstand transport and various environmental conditions Known and acceptable accuracy and precision of measurements
Logistics	Ability to conduct measurements in a controlled environment Transporting equipment Ensuring access to compatible and reliable power sources Allocating sufficient time for set-up, calibrations, and testing
TDEE with DLW	
Athletes	Ability of athletes to provide urine samples (e.g., limited by time, training, and potentially, dehydration)
Researcher	Ability to follow DLW dosing and urine collection/storage protocols Need clear communication on who will lead sample collection, storage, shipping, and analyses Need for highly-trained researchers for analyses of isotope concentrations
Equipment	Need for highly technical, costly, and specific analytical techniques (e.g., isotope ratio mass spectrometry) only available at a few institutions worldwide
Logistics	Sample collection procedures (especially on days with multiple urine samples), including when and where samples will be collected and stored Shipping requirements (e.g. procuring shipping materials, country-specific regulations on biological samples)

DLW, doubly labeled water

## Field-Based Female Athlete’s Methodological Considerations

Female athletes have been underrepresented in sports science research, as many studies typically recruit males and extrapolate findings to females with limited regard to potential sex-based differences that may influence physiological and performance outcomes [157–161]. Many biological sex-based differences potentially arise from monthly menstrual cycle fluctuations in sex hormones [luteinizing hormone (LH), follicle-stimulating hormone (FSH), estrogen (estradiol; E2), and progesterone (P4)] that occur from the onset of puberty until menopause [162]. Importantly, the menstrual cycle and its various phases can be unique to each female, and even the same individual can differ month to month [18]. These sex-based monthly menstrual cycle fluctuations in sex hormones can not only impact physiological outcomes but can also potentially have an impact on nutritional/energetic, biomechanical, musculoskeletal, cognitive, and psychosocial factors.

Determination of menstrual status/phase in sports science research serves many purposes. In addition to providing medical information about an athlete’s hypothalamic–pituitary–adrenal (HPA) axis status (energy/health/menstrual dysfunctions) and study inclusion suitability, it can be used to confirm time points for data collection or to characterize sex hormone concentrations at the time of testing. Elliott-Sale et al. [163] provide a list of menstrual cycle iterations and methodological requirements ranging from *apparently* regular menses (eumenorrhea; naturally menstruating but potentially anovulatory) to irregular (oligomenorrhea) or absent cycles (primary and secondary amenorrhea). It is important to note that not all female athletes with self-reported regular monthly periods are eumenorrheic with evidence of LH surge and correct hormonal profile. Indeed, up to 50% of female athletes with seemingly regular cycles experience abnormalities such as anovulation or luteal phase defects [164, 165]. For example, a study comparing sedentary and exercising females with natural menstrual cycles reported that, while 90% of sedentary females had ovulatory cycles, only 45% of exercising females experienced ovulation; the remaining were classified with either a luteal phase defect or anovulation [164]. Clinical conditions [e.g., polycystic ovary syndrome (PCOS), hypo/hyperthyroidism] should also be considered. While pregnant or breastfeeding athletes are often excluded from sports science research, specific studies focusing on these athletes are needed to increase our knowledge and understanding of the impact of the distinct hormonal milieus characterized by these conditions.

Approximately 50–60% of the athletic premenopausal female populations from Western Europe, Australia, and North America use some form of contraception [166–168]. Contraception alternatives range from hormonal and nonhormonal, orally administered (“the pill”), to patches and intrauterine devices (IUDs)[168]. In particular, all oral contraceptive pills suppress ovulation, with some resulting in about four- to eightfold higher progestin exposure when compared with endogenous progesterone, which may have far reaching impacts on physiology [169]. However, it does not appear that contraceptives impact performance in athletes [170]. These are several examples of why further research is required to advance the methodological assessment of athletes using contraception [167].

Methods to characterize menstrual status in field research vary in terms of athlete burden and cost of implementation (Table 7). Our experiences successfully implementing menstrual status classification in studies of free-living, high-performance athletes [18] support the recommendation to make this a requirement in all research involving female athletes. The minimum standard should involve cycle tracking with confirmed ovulation. Meanwhile, direct measurement of hormonal status is considered ideal and should be undertaken in studies where hormonal milieu, menstrual cycle phase/status, or sex differences are the primary objective [171]. Unfortunately, protocols that *control* for menstrual cycle phase in field-based research (i.e. standardize the phase in which all athletes are tested) can be difficult. This is especially true in study designs involving group training and/or performance measures (e.g., competition or training camps), as the logistics of assembling elite athletes and testing schedules are already challenging. Indeed, in many circumstances, methodological considerations are likely to be dictated by study design, primary study aims, and resources.

**Table 7:** Framework for female field-based interventions for monitoring and assessment of the endocrine milieu

Outcome	Methodology	Athlete burden	Cost	Comments
*Menstrual cycle tracking, menstrual cycle status assessments*^a^				
**Cycle tracking** (cycle length, duration of bleeding)	**What:** Apps^b^ (e.g., Flo, Clue, Garmin) Calendar **When:** Calendar tracking throughout the cycle	Relatively low to Medium	Low	Allows for assessment of cycle length and duration of bleeding Easy to implement in the field Ideally implemented as a 3-month pre-tracking phase prior to the actual data collection period to gain an understanding of individual cycle characteristics and variability
**Ovulation** (day of ovulation)	**What:** Urinary LH strips (e.g., Clearblue) **When:** On day ~ 8 until positive LH surge	Relatively low	Relatively low to Medium (depending on brand)	Confirms LH peak and assists in determining timing of sex hormone (and other markers) assessments Relatively easy to implement in the field (NOTE: not all LH peaks confirm ovulation, as anovulatory cycles can occur in the presence of an LH surge. Thus, a sustained (~ 3 day) increase (~ 0.5 ºC) in basal body temperature along with LH detection can further confirm ovulation [1])
**Sex hormone concentrations** (physiological concentrations of sex hormones throughout the cycle can be used to plot the cycle characteristics)	**What:** Urinary (e.g., Oova); Saliva samples Venous blood samples or; Fingerprick blood samples For the measurement of LH, FSH, E2, P4 **When:** Daily or at 4 time points throughout the cycle (e.g., days ~ 2, ~ 14, ~ 15, ~ 21)	Medium to high depending on whether home or laboratory-based assessments or whether measured via blood or saliva sampling	High	Required to confirm menstrual cycle status and hormonal milieu May be challenging to implement in the field; venous blood sampling relies on access to a laboratory or processing and storage of blood samples, likely requiring additional facilities Use of technology such as Oova home test kits provides an alternative solution to the field environment Important to consider test validity prior to implementation
*Other female specific assessments*				
**Menstrual cycle related symptoms** (can be used to assess negative and positive symptoms, management strategies and effects on training, recovery, sleep, performance associated with the cycle)	**What:** Self-report surveys (e.g., [18]) **When:** Based on study aims	Low	Low	Surveys online or on paper, based on desired outcome Can be utilized to provide information on biopsychosocial considerations around female athlete specific issues Easy to implement in the field ^Can represent a burden to the athlete if long or implemented frequently

LH, luteinizing hormone; FSH, follicle-stimulating hormone; E2, estrogen; P4, progesterone

^a^Can be assessed in athletes with irregular or absent cycles or those using hormonal or nonhormonal contraception but with use of different time points. For detailed methodology guidelines please refer to [163, 173]

^b^Section 4 highlights the need for very careful consideration regarding who has information access and subject confidentiality regarding third-party “apps,” which is especially true of period/menstrual tracking apps that have many benefits, but also potentially many drawbacks, including privacy and confidentiality of personal medical information [76, 77]

## Conclusions

Descriptive field-based research is often the only approach suitable for characterizing the extreme demands of elite training and competition, offering insights into human capacity limits [3, 12, 172]. Traditionally, comprehensive, field-based data collection, at either training camps or competitions are rare opportunities conducted over discrete timelines. Therefore, compared with most laboratory-based studies, these once-in-a-lifetime opportunities usually require a large research staff to efficiently execute, with minimal distractions to teams and athletes. For example, we recently undertook a multi-disciplinary field-based research study over a 6-day ultramarathon that required ~ 1.5 years of planning, with 29 researchers, split across 6 main sub-discipline teams requiring ~ 2400 h of accumulated work over a scant 12-day period (when including ~ 3 days of pre-event and post-event testing) that produced > 1 terabytes of data (findings and publications to follow). These types of intensive, singular field-based studies usually result in numerous publications, which can sometimes be criticized for overlap/duplication of data across the various publications but are also required to transparently report all relevant findings.

Ultimately, the ideal scientific design, execution, and interpretation of field-based research studies, along with the successful in-field (competition and/or training) implementation of the results, require: (1) researchers who have intimate knowledge of a sport/event; (2) the trust of the athletes, teams, and coaches; (3) exceptional logistical and organizational skills; and (4) rigorous scientific expertise to apply the various field-based methodologies highlighted above. The integration of these four vital prerequisites are the cornerstones of success for sport field-based research.

## Supplementary Information

Below is the link to the electronic supplementary material.

Supplementary file1 (PDF 59 KB)
