# Supplementary material for: Integrative Field-Based Health and Performance Research: A Narrative Review on Experimental Methods and Logistics to Conduct Competition and Training Camp Studies in Athletes
Source: Sports Med. 2025 Apr 21;55(6):1377–403. doi: 10.1007/s40279-025-02227-0 (PMC12152029; doi:10.1007/s40279-025-02227-0)
Supplement: Supplementary file 1 — Supplementary file1 (PDF 59 KB) [file 40279_2025_2227_MOESM1_ESM.pdf]

Narrative Review

Integrative field-based health and performance research: a narrative review on experimental methods and logistics to conduct competition and training camp studies in athletes

Trent Stellingwerff[1,2,3], Louise M. Burke[4], Hannah G. Caldwell[5,6], Robert J. Gathercole[7], Chris J. McNeil[5], Christopher Napier[8], Sarah A. Purcell[5,9], Susan Boegman[1], Elizabeth Johnson[1], Sharleen D. Hoar[1], Alexandra M. Coates[8], Erica V. Bennett[3], Alannah K. A. McKay[4], Ida. A Heikura[1,2], Michael J. Joyner[10] and Jamie F. Burr[11]

[1]*Canadian Sport Institute - Pacific, Victoria, British Columbia, Canada;*
[2]*Exercise Science, Physical & Health Education, University of Victoria British Columbia, Canada;*
[3]*School of Kinesiology, The University of British Columbia, Vancouver, British Columbia, Canada;*
[4]*Mary Mackillop Institute for Health Research, Australian Catholic University, Melbourne, Victoria, Australia;*
[5]*School of Health and Exercise Sciences, The University of British Columbia - Okanagan campus, Kelowna, British Columbia, Canada;*
[6]*The August Krogh Section for Human Physiology, Department of Nutrition, Exercise and Sports, University of Copenhagen, Copenhagen, Denmark;*
[7]*Product Innovation Team, Lululemon Athletica, Vancouver, British Columbia, Canada;*
[8]*Department of Biomedical Physiology and Kinesiology, Simon Fraser University, Vancouver, British Columbia, Canada;*
[9] *Centre for Chronic Disease Prevention and Management, Southern Medical Program, Department of Medicine, The University of British Columbia, Kelowna, British Columbia;*
[10]*Department of Anesthesiology and Perioperative Medicine, Mayo Clinic, Rochester, Minnesota, USA;*
[11]*Human Health and Nutritional Sciences, University of Guelph, Guelph, Ontario, Canada.*

Running title: *Field-based competition and training camp research*

Journal: *Sports Medicine:*

Address for correspondence:

Dr. Trent Stellingwerff

Canadian Sport Institute - Pacific

Pacific Institute for Sport Excellence

4371 Interurban Road

Victoria, British Columbia, Canada, V9E 2C5

Mobile: +1 250.208.6674

Email: tstellingwerff@csipacific.ca

Supplementary Material Table 1. *A priori* publication planning template.

**Proposed title**	
**Theme**	
**Target journal**	
**Main Data Sets (e.g. bloods vs. biomech vs. EI vs. DLW)**	
**Variables - Bolded = key outcome variables // non-bolded - sub outcome variables**	
**Sample size / data issues / limitations**	
**Lead author**	
**Co-authors**	
**Acknowledgements**	
**Progress**	
**Outstanding**	

Examples of statistical analyses or examples of figures / tables that could be ideal for this paper.

Supplementary Table 2. A checklist for building a portable physiological testing kit for research purposes

**Equipment**	
☐ Heart rate straps<br>☐ Heart rate watch<br>☐ RPE scale<br>☐ Handheld infrared thermometers<br>☐ Specialized equipment/wearables	☐ Clipboards<br>☐ Pens<br>☐ Body mass scales<br>☐ Data capture sheets<br>☐ Laptops and/or tablets
**Biohazardous materials/blood sampling**	
☐ Biohazard bags<br>☐ Sharps containers (where needed)<br>☐ Bench roll/Bench pads<br>☐ Tissues<br>☐ Alcohol swabs<br>☐ Hand Sanitizer<br>☐ Gloves (in appropriate sizes)<br>☐ Lancets	☐ Portable blood analyzers<br>☐ Test strips for analyzers<br>☐ Calibration strips for analyzers<br>☐ Micropore tape<br>☐ Band aids<br>☐ Disinfectant<br>☐ Face masks/PPE
**General items**	
☐ Spare pens and markers<br>☐ Spare batteries<br>☐ Tools (e.g. Allan keys, spanner)<br>☐ Duct tape<br>☐ Nutrition (e.g., water bottles, carbohydrate gels, snacks)<br>☐ Consent forms	☐ Charging cables<br>☐ Scissors<br>☐ Stopwatches<br>☐ Refractometers<br>☐ Towels<br>☐ Team communication devices (radios, cell phones)
